# Circular RNA TAF4B targeting MFN2 accelerates cell growth in bladder cancer through p27 depression and AKT activation

**DOI:** 10.3389/fimmu.2024.1477196

**Published:** 2024-10-04

**Authors:** Xiaoting Zhang, Jia Xu, Guangzhen Zhuang, Yiting Wang, Xiaofeng Li, Xiaohui Zhu

**Affiliations:** ^1^ Central Laboratory, Shenzhen Bao’an District Songgang People’s Hospital, Shenzhen, China; ^2^ Department of Medicine and Health, The University of Sydney, Sydney, NSW, Australia; ^3^ College of Pharmacy, Shenzhen Technology University, Shenzhen, China; ^4^ Department of Laboratory Medicine, Peking University Shenzhen Hospital, Shenzhen, China

**Keywords:** bladder cancer, circTAF4B, MFN2, p27, Cell Cycle, AKT signaling

## Abstract

**Introduction:**

Bladder cancer (BCa) is a common malignancy in the urinary tract. It has high recurrence rates and often requires microscopic examination, which presents significant challenges in clinical treatment. Previous research has shown that circular TAF4B (circTAF4B) is significantly upregulated in BCa and is associated with a poor prognosis. However, the specific targets and molecular mechanisms by which circTAF4B functions in BCa are still not well - understood.

**Methods:**

In this study, an RNA pull - down assay and mass spectrometry were utilized to identify MFN2 as a binding protein of circTAF4B. Additionally, siRNA was used to silence MFN2 to observe the amplification of the inhibitory effects of circTAF4B overexpression on cell growth and migration in BCa cells. Moreover, circTAF4B shRNA lentiviral particles were employed to study their impact on BCa progression by examining the regulation of p27 and the blocking of AKT signaling.

**Results:**

It was found that MFN2 is a binding protein of circTAF4B. Silencing MFN2 with siRNA enhanced the inhibitory effects of circTAF4B overexpression on cell growth and migration in BCa cells. Also, circTAF4B shRNA lentiviral particles inhibited BCa progression by upregulating p27 and blocking AKT signaling.

**Discussion:**

In conclusion, the physical binding of circTAF4B to MFN2 is a crucial process in the tumorigenesis and progression of BCa. Targeting circTAF4B or its complexes may have potential as a therapeutic strategy for BCa diagnosis and treatment.

## 1 Introduction

Bladder cancer (BCa) ranks as the ninth most common cancer in terms of incidence and thirteenth in mortality worldwide (1). The American Cancer Society reported 82,290 new cases and 16,710 deaths in the United States in 2023, with male cases outnumbering female ones by more than two to one. Among male-specific tumors, BCa is the fourth most prevalent in incidence (62,420 cases) and eighth in mortality (12,160 deaths) (2). Microscopy remains the gold standard for diagnosing BCa in clinical practice (3). Despite a notable yearly decline in urinary BCa rates over the past decade (4), the disease’s high recurrence rate and the need for frequent microscopic examinations contribute significantly to patient discomfort and elevated treatments (5). Consequently, a comprehensive and thorough investigation into the pathogenesis of BCa holds immense clinical and social value, potentially easing patient suffering and improving treatment outcomes.

According to the criteria set by the WHO International Society of Urological Pathology, BCa is classified into non-muscle-invasive bladder cancer (NMIBC) and muscle-invasive bladder cancer (MIBC) (6).An increasing body of clinic evidence indicates that cell proliferation is closely associated with lymphatic metastasis in BCa, which is modulated by circular RNAs (circRNAs) (7). CircRNAs, possess a closed circular structure without a 5′ cap and a 3′ poly(A) tail, which grants them greater stability than linear RNAs (8, 9). As research into circRNAs deepens, numerous studies have explored their relationship with BCa. For example, circ_0000851 binds directly to miR-1183, enhancing the expression of its target gene PDK1, which promotes BCa cell proliferation and migration by activating the PDK1/p-AKT signaling pathway (10). Other circRNAs, such as circATIC, foster BCa development by modulating CDK6 expression (11), while circTCF25 and CircZFR promotes BCa cell proliferation and migration through the miR-107/FOX-K1 axis and by acting as a sponge of miR-377 to enhance parental gene ZEB2 expression (12, 13). Conversely, circSHPRH inhibits BCa cell proliferation by targeting miR-942 and upregulating BARX2 expression (14).

TATA-box binding protein-associated factor 4b (TAF4B) is a cell type-specific TBP-associated transcriptional factor found on chromosome 18’s long arm (q) of (15). It combines with TAF12 to facilitate the binding of transcription factor IID (TFIID) with core promoter elements (16). Specifically, its physical interactions with various proteins to form complexes is essential for initiating and regulating gene transcription. Dysregulation of the TAF4B gene is implicated in diseases and abnormal cellular processes such as uncontrolled meiosis, chromatin modification disorders, and inappropriate expression of X-linked genes (17). TAF4B also plays a role in follicular development in pigs, regulated by follicle-stimulating hormone (FSH) (18). However, its specific functions vary by context, as ongoing research across different fields reveals.

CircTAF4B (hsa_circ_0047322, http://www.circbase.org/) arises from the alternative back-splicing of exons 10-13 of the TAF4B gene, resulting in a 484 nt circRNA (19). Mediated by the spliceosome machinery, this unconventional splicing event connects the downstream end of exon 13 to the upstream end of exon 10, bypassing intervening exons and resulting in circularization of the exons to form circTAF4B. Previously, we identified circTAF4B as significantly upregulated in BCa, associated with poor prognosis. Its inhibition disrupted cell growth, metastasis, and epithelial-mesenchymal transition (EMT) by sponging miR-1298-5p and promoting transforming growth factor A (TGFA) expression in BCa cells (20). However, the precise targets and molecular mechanisms of circTAF4B in BCa remain to be fully understood.

In this study, we first identified mitochondrial fusion protein 2 (MFN2) as one of the binding proteins of circTAF4B. The downregulation of MFN2 enhanced the inhibitory effects of circTAF4B on BCa cell growth and migration. Mechanistically, the inhibition of circTAF4B suppressed BCa progression through p27-mediated cell cycle arrest and AKT signaling blocking. Therefore, thorough elucidation of the role and molecular mechanisms of circTAF4B holds significance clinical relevance for treating BCa and potentially other tumors.

## 2 Materials and methods

### 2.1 Reagents

Dulbecco’s modified Eagle’s medium (Cat#11965118, Gibco, Grand Island, NY, United States) and fetal bovine serum (Cat#10099141C, Gibco, Grand Island, NY, United States) were obtained from Thermo Fisher Scientific Inc. The following antibodies were used to targeting specific markers in western blotting: p27 (Cat# 3686, Cell Signaling Technology, Danvers, MA, United States), cyclin D1 (Cat# 2978, Cell Signaling Technology, Danvers, MA, United States), cyclin D3 (Cat# 2936, Cell Signaling Technology, Danvers, MA, United States), Phospho-EGFR (Cat# 4407, Cell Signaling Technology, Danvers, MA, United States), EGFR (Cat# 71655, Cell Signaling Technology, Danvers, MA, United States), Phospho-AKT (Cat# 9271, Cell Signaling Technology, Danvers, MA, United States), AKT (Cat# 9272, Cell Signaling Technology, Danvers, MA, United States), GAPDH (Cat# 5174, Cell Signaling Technology, Danvers, MA, United States), rabbit IgG (Cat# 3900, Cell Signaling Technology, Danvers, MA, United States), and mouse IgG (Cat# 5415, Cell Signaling Technology, Danvers, MA, United States) from Cell Signaling Technology; eIF4A3 (17504-1-AP, Proteintech, Wuhan, Hubei, China), DDX10 (17857-1-AP, Proteintech, Wuhan, Hubei, China), RINT1 (14567-1-AP, Proteintech, Wuhan, Hubei, China), and MFN2 (12186-1-AP, Proteintech, Wuhan, Hubei, China) from Wuhan Sanying BioTech. Co. Ltd.; Raf1 (R25538, ZenBio, Chengdu, Sichuan, China) was purchased from ZEN-BIOSCIENCE Co. Ltd.; and MEF2 (ab170946, Abcam, Cambridge, CB2 0AX, UK) was form Abcam Limited.

### 2.2 Cell lines

The following cell lines were used: T24 (TCH-C352), 5637 (TCH-C104), SW780 (TCH-C344), and J82 (TCH-C221) from Haixing Biosciences Co., Ltd. (Suzhou, China); SV-HUC1 (CRL-9520) and HEK 293T (CRL-3216) from American Type Culture Collection (ATCC, Manassas, VA, USA).

### 2.3 Primers, siRNAs, and lentiviral particles

All primers (**Table 1**) and siRNAs (**Table 2**) were synthesized by GenePharma Co., Ltd. (Suzhou, China). U2 snRNA and β-actin were used as internal references. CircTAF4B shRNA lentiviral particles were produced by GenePharma Co., Ltd. CircTAF4B overexpression lentiviral particles were prepared in our laboratory.

**Table 1:** qPCR primers for circTAF4B and detected genes.

	Name	Forward primer (5'-3')	Reverse primer (5'-3')
Divergent primers	circTAF4B	TAAGGCAGCCAAGAGTCGTT	TAAGGTTGACCCCTGCCATA
mRNA	TAF4B	TAAGGCAGCCAAGAGTCGTT	AGCTGCAAGAGCTGTGAGATTA
	β-actin	ACAGAGCCTCGCCTTTGCCGAT	CTTGCACATGCCGGAGCCGTT
snRNA	U2 snRNA	CTTCTCGGCCTTTTGGCTAAGA	AGTGGACGGAGCAAGCTCCTAT

**Table 2:** siRNAs and shRNAs.

Name	Sense (5'-3')	Antisense (5'-3')
sh_circTAF4B	GAAAGCCAAAGAGAGATGAGG	
NC	UUCUCCGAACGUGUCACGUdTdT	ACGUGACACGUUCGGAGAAdTdT
si_MFN2	GGAAGAGCACCGUGAUCAAdTdT	UUGAUCACGGUGCUCUUCCdTdT

### 2.4 qPCR detection

Total RNA was extracted from the samples and reverse-transcribed into cDNA. In a PCR tube or a qPCR well, the following components were combined: cDNA template, forward and reverse primers (β-actin as a positive control), and qPCR reaction mix. The reaction setup was placed in a qPCR instrument and subjected to the following cycling steps: initial denaturation at a high temperature, followed by multiple cycles of denaturation, annealing, and extension, with fluorescence detection at each cycle. After completion, cycle threshold (Ct) values were calculated and standard curves were generated for absolute or relative quantification.

### 2.5 Nuclear/cytoplasmic RNA isolation

The nuclear and cytoplasmic RNA was isolated using a Kit (Cat. 21000, Norgen, Biotek Corp., ON, Canada). Following the separation of nuclear and cytoplasmic RNA as the manufacturer’s instructions, the expression of the target gene was quantified by qRT-PCR with β-actin and U2 serving as controls.

### 2.6 MTS assay

Cells transfected with indicated siRNAs and plasmids were seeded into 96-well plates at an appropriate density. At 0, 24, 48, 72, 96, and 120 hours, the fresh medium was replaced with MTS reagent (v/v = 9:1) according to the manufacturer’s instructions. Absorbance was measured after incubation for 3 hours 37° in 5% CO_2_.

### 2.7 Colony formation

Cells transfected with specified siRNAs and plasmids were seeded into 24-well plates at 500-1000 cells/well. Cells were incubated at 37° in 5% CO_2_ for 10-14 days to allow for growth and colony formation. Post-incubation, cells were fixed with 4% paraformaldehyde for 15 min and stained with 0.1% crystal violet for 15 min. Colonies were counted under a microscope or by alternative methods.

### 2.8 RNA pull down

Biotinylated circTAF4B and scramble probes were synthesized by RiboBio Co., Ltd. (Guangzhou, China). HEK 293T cells transfected with pLC5-circTAF4B plasmids were lysed using NP-40 lysis buffer (Cat. P0013F, Beyotime, Shanghai, China), supplemented with RNase inhibitor (Cat. 3335399001, Roche, Basel, Switzerland) and protease inhibitor cocktail (Cat. 4693116001, Roche, Basel, Switzerland). Following centrifugation, the supernatant of lysates was incubated with 2 μg of the corresponding probes at room temperature for 4 hours. Subsequently, 50 μL of streptavidin-coated magnetic beads (Cat:65306, Invitrogen, Vilnius, Lithuania) were added to the tubes and rotated for 30 min at room temperature. The magnetic bead-probe-circTAF4B-protein complexes were then analyzed by mass spectrometry, reserving 20 μL of the complex for Western blot analysis.

### 2.9 Western blot

Proteins separated by SDS-PAGE were transferred to a PVDF membrane. The membrane was then blocked and incubated with primary and secondary antibodies, followed by detection using with chemiluminescence to visualize protein bands.

### 2.10 Cell cycle analysis

Cells were transfected with specified siRNAs and plasmids for 48 hours, collected and washed with PBS, and fixed with 70% ethanol at 4° overnight. Subsequently, cells were stained with propidium iodide (PI) for 15 min in the dark and analyzed using flow cytometry to determine the cellular distribution in various phases.

### 2.11 Wound healing assay

Cells transfected with specified siRNAs and plasmids were seeded in 6-well plates at 2×10^5^ cells/well. A scratch was created on the cell monolayer. The cells were then intubated at 37° in 5% CO_2_. The closure of the wound was monitored and measured at specified time points (0, 6, 12, 24, 36, 48 hours) to assess cell migration and healing abilities.

### 2.12 Statistical analyses

Statistical analyses were performed using GraphPad Prism 8.0 (GraphPad Inc., La Jolla, CA, USA). Inter-group differences were evaluated using Student’s t-test or one-way ANOVA, as appropriate. *p <* 0.05 was statistically significant.

## 3 Results

### 3.1 Expression and localization of circTAF4B and *TAF4B* gene

**Figure 1:**
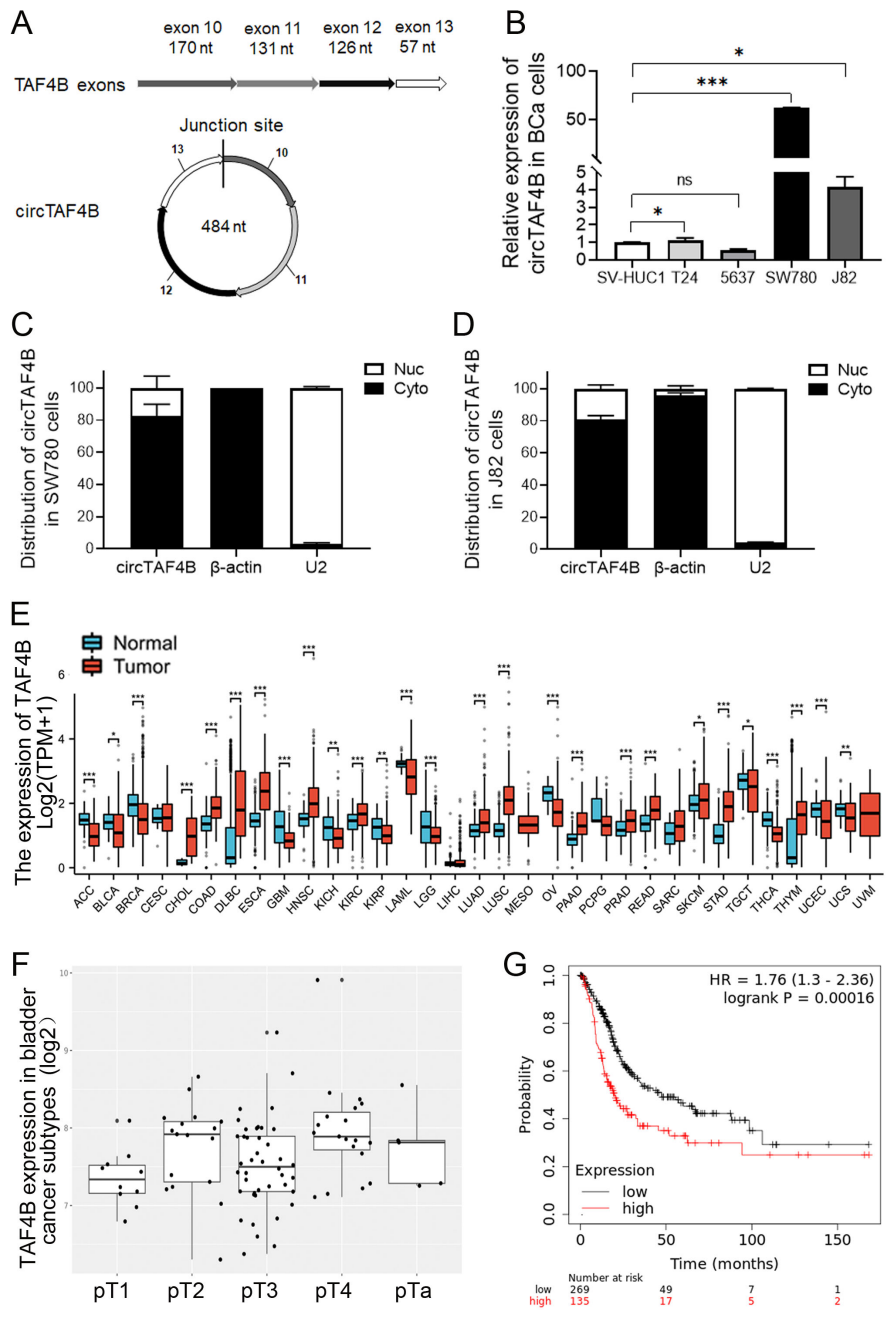
Characterization of circTAF4B and expression patterns of TAF4B mRNA in BCa cell lines and human GC tissues. **(A)** Verification of the junction site of circTAF4B by Sanger sequencing; **(B)** RT-qPCR analysis of circTAF4B expression levels in BCa cell lines, three independent experiments, two-group differences were evaluated using Student’s t-test, *p < 0.05, **p < 0.01, ***p < 0.001; **(C, D)** RT-qPCR analysis of the distribution of circTAF4B in the nuclei and cytoplasm of SW780 and J82 cells, with β-actin and U2 as cytoplasmic and nuclear controls, respectively, and three independent experiments were conducted; **(E)** Expression of the *TAF4B* gene in normal and primary tumor samples analyzed using the GENT2 platform; **(F)** Stage-wise expression of the *TAF4B* gene across BCa subtypes analyzed using the GENT2 platform; and **(G)** K-M curve depicting the association between *TAF4B* gene expression levels and overall survival (OS) of BCa patients analyzed using the Kaplan-Meier Plotter platform.

Utilizing circRNA sequencing data sourced from the reputable databases circBase (http://www.circbase.org) and circBank (http://www.circbank.cn), we discerned an ectopic circular RNA, circTAF4B, with a length of 484 nucleotides, which is derived from exons 10 to 13 of the *TAF4B* gene (**Figure 1A**). Through qPCR validation, we confirmed that circTAF4B (hsa_circ_0047322, http://www.circbase.org/) is significantly upregulated in BCa cell lines, with particularly high expression levels in the SW780 and J82 cell lines (**Figure 1B**). To evaluate the intracellular distribution of circTAF4B, we conducted nuclear and cytoplasmic RNA extractions, followed by qPCR analysis in the SW780 and J82 cells. Our findings indicated a predominant cytoplasmic localization of circTAF4B (**Figures 1C, D**). This observation intimates that circTAF4B may modulate the stability and translation of cytoplasmic mRNAs and potentially perturb signaling pathways. Given that circRNAs often manifest biological functions that are closely associated with their parental genes, a sophisticated regulatory network is implicated. Employing the Xiantao platform (xiantaozi.com), our observations revealed that TAF4B expression was generally subdued across a spectrum of cancer tissues, including BCa (**Figure 1E**), and displayed variability in expression levels correlating with different stages and subtypes of bladder cancer (**Figure 1F**). Further analysis via the Kaplan-Meier plotter platform (http://kmplot.com/analysis/) suggested a correlation between lower TAF4B expression and improved overall survival (OS) rates among patients, in contrast to those with elevated expression levels (**Figure 1G**).

### 3.2 circTAF4B promoting cell growth and colony formation in BCa cells

**Figure 2:**
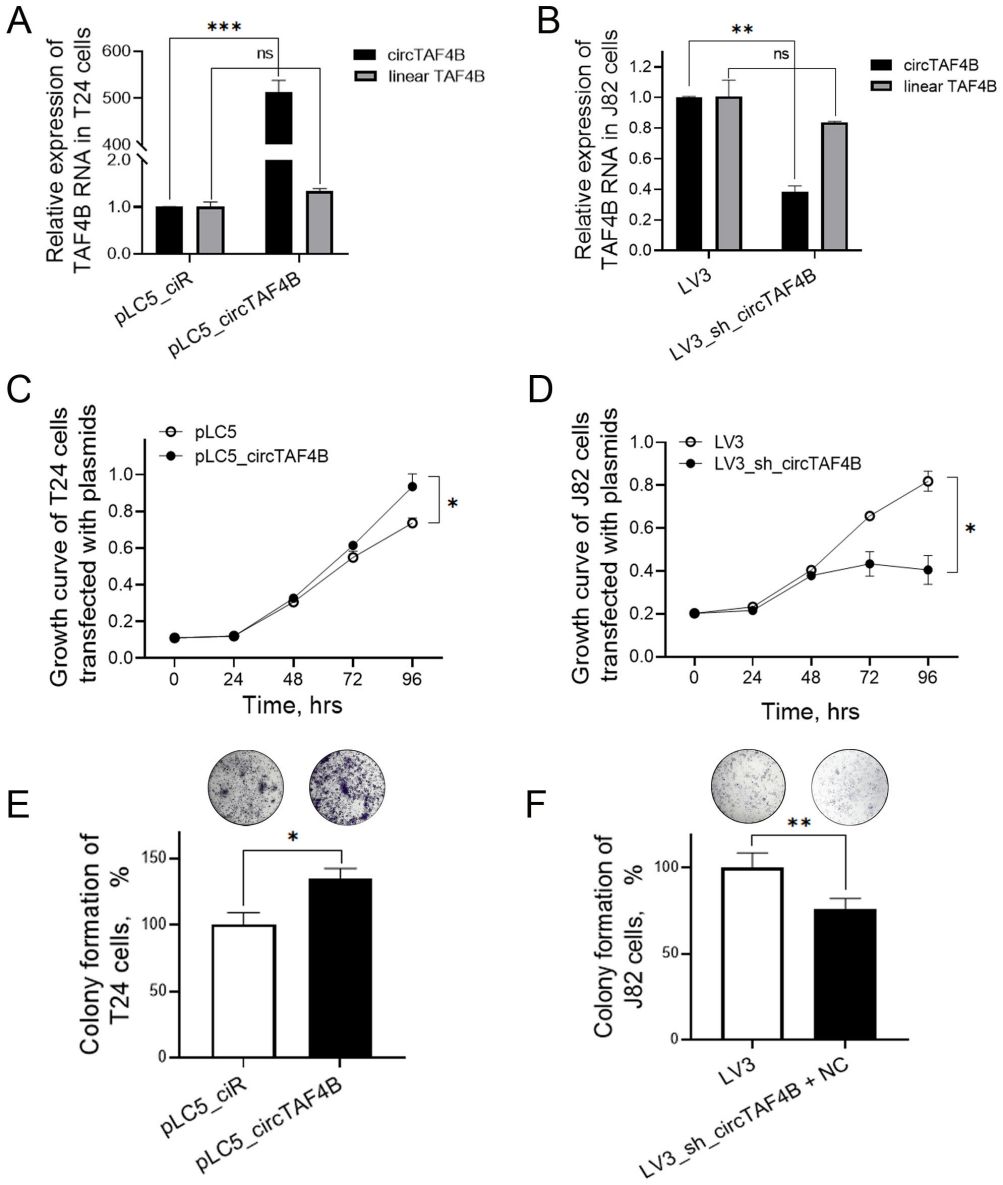
Effects of circTAF4B on cell growth in T24 and J82 cells. **(A)** Forced expression of circTAF4B did not affect TAF4B mRNA levels; **(B)** Knockdown of circTAF4B did not affect TAF4B mRNA levels; **(C)** Forced expression of circTAF4B promoted cell proliferation in T24 cells; **(D)** Knockdown of circTAF4B suppressed cell proliferation in J82 cells; **(E)** Forced expression of circTAF4B promoted colony formation in T24 cells; and **(F)** Knockdown of circTAF4B inhibited colony formation in J82 cells. At least three independent experiments were conducted, and two groups were statistically analyzed with Student’s t-test, *p < 0.05, **p < 0.01, ***p < 0.001.

To investigate circTAF4B’s role in BCa, we developed an overexpression plasmid (pLC5_ circTAF4B) and a knockdown lentiviral system (LV3_sh_circTAF4B). Overexpression of circTAF4B sharply increased circTAF4B levels in T24 cells, and did not alter *TAF4B* mRNA levels (**Figure 2A**). While knockdown of circTAF4B significantly decreased circTAF4B levels in J82 cells, and did not alter *TAF4B* mRNA levels (**Figure 2B**). MTS assays demonstrated that forced-expression of circTAF4B promoted cell proliferation in T24 cells (**Figure 2C**), whereas knockdown of circTAF4B inhibited proliferation in J82 cells (**Figure 2D**). Colony formation assays showed increased colony numbers following circTAF4B overexpression in T24 cells (**Figure 2E**) and reduced colony numbers following circTAF4B knockdown in J82 cells (**Figure 2F**).

### 3.3 Identification of MFN2 as a circTAF4B binding protein

**Figure 3:**
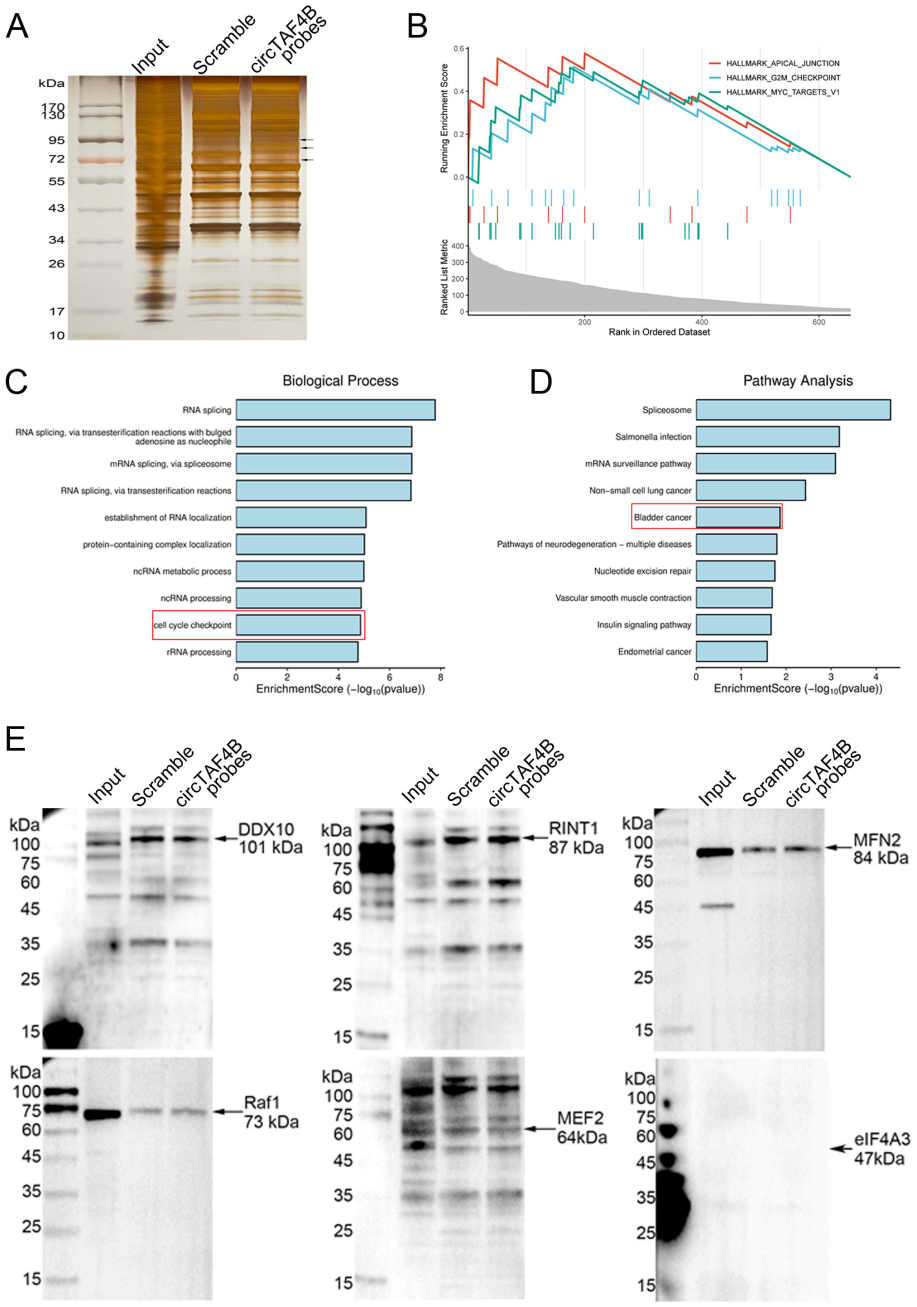
Identification of MFN2 as a circTAF4B binding protein. **(A)** CircRNA pull-down assay using circTAF4B and scramble probes followed by silver staining, two independent experiments were conducted; **(B–D)** Identification of proteins pulled down by circTAF4B probes *via* mass spectrometry. 656 proteins were selected based on an iBAQ circTAF4B probe/scramble probe ratio of > 5.0. Gene set enrichment analysis (GSEA), Gene Ontology (GO), and KEGG pathway analysis of the 656 proteins; and **(E)** Western blot confirmation of MFN2 as a binding partner of circTAF4B, at least three independent experiments were conducted.

To elucidate the molecular mechanisms underlying circTAF4B’s function in BCa, we performed RNA pull-down assay using biotinylated circTAF4B probes in HEK 293T cells transfected with pLC5_circTAF4B plasmids in order to capture circTAF4B binding proteins (**Figure 3A**). Subsequent mass spectrometry (MS) analysis identified 656 proteins. Gene set enrichment analysis (GSEA), Gene Ontology (GO), and KEGG pathway analysis indicated that these proteins could modulate cell proliferation and the cell cycle in BCa (**Figures 3B–D**). Through further analysis based on the probe/scramble ratio and signal intensity of the MS-identified proteins, we narrowed down six candidates (DDX10, RINT1, MFN2, Raf1, MEF2, eIF4A3). Western blotting subsequently confirmed MFN2 as a circTAF4B binding protein (**Figure 3E**).

### 3.4 Impact of MFN2 knockdown on circTAF4B shRNA’s inhibitory effects

**Figure 4:**
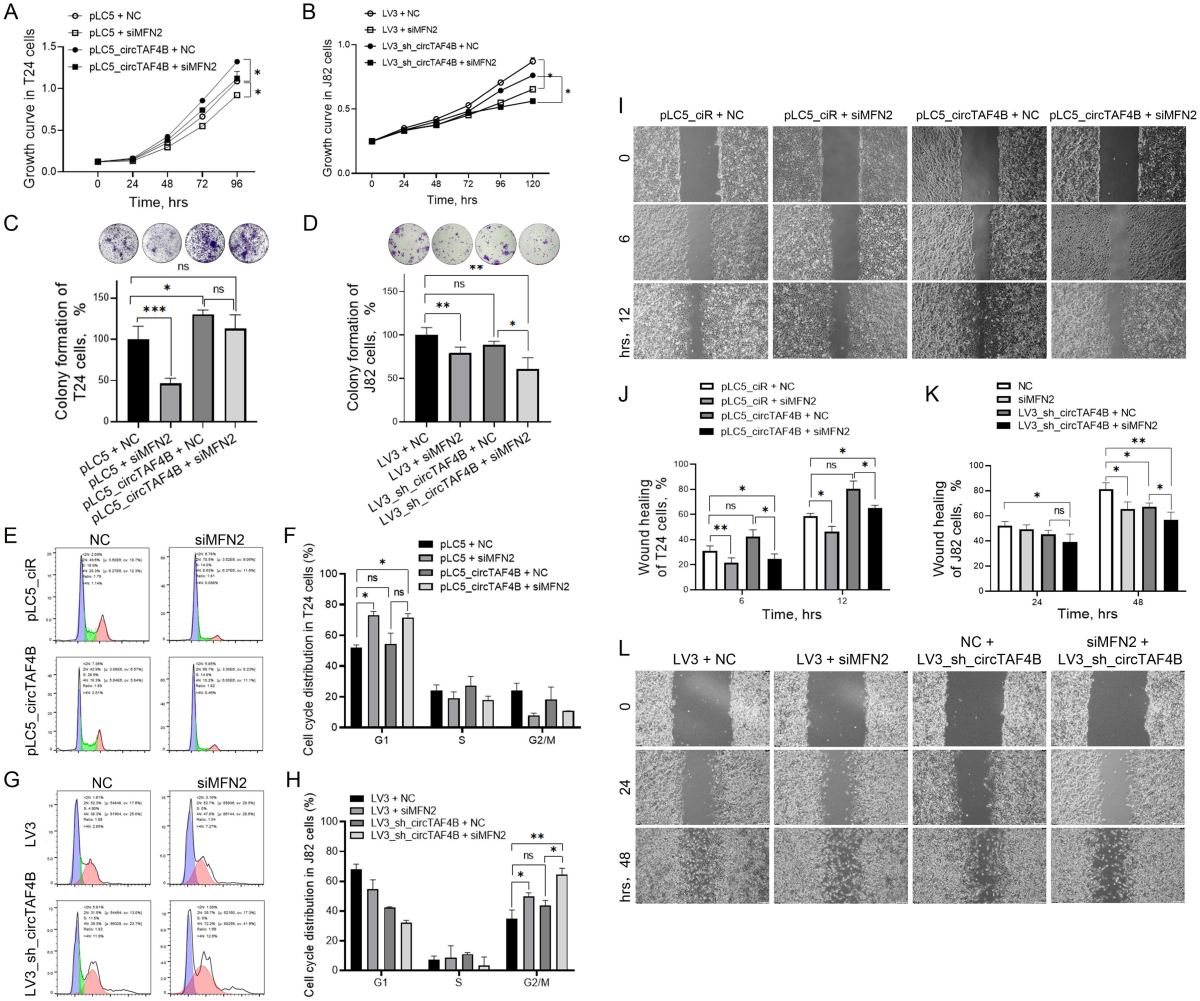
Knockdown of MFN2 enhanced circTAF4B shRNA’s inhibitory effects on cell growth, colony formation, and cell migration in BCa cells. **(A)** Forced expression of circTAF4B promoted cell proliferation, while MFN2 siRNAs inhibited cell proliferation in T24 cells; **(B)** Knockdown of MFN2 enhanced circTAF4B shRNA’s inhibitory effects on cell growth in J82 cells; **(C)** Forced expression of circTAF4B promoted colony formation, while MFN2 siRNAs inhibited colony formation in T24 cells; **(D)** Knockdown of MFN2 enhanced circTAF4B shRNA’s inhibitory effects on colony formation in J82 cells; **(E, F)** Forced expression of circTAF4B did not affect cell cycle significantly, while MFN2 siRNAs blocked cell cycle in T24 cells; **(G, H)** Knockdown of MFN2 enhanced G2/M cell cycle arrest of circTAF4B shRNA in J82 cells; **(I, J)** Forced expression of circTAF4B promoted cell migration, while MFN2 siRNAs inhibited cell migration in T24 cells; and **(K, L)** Knockdown of MFN2 enhanced circTAF4B shRNA’s inhibitory effects on cell migration in J82 cells. At least three independent experiments were conducted, and two groups were statistically analyzed with Student’s t-test, Inter-group differences between more than two groups were evaluated using one-way ANOVA, *p < 0.05, **p < 0.01, ***p < 0.001.

To explore how the circTAF4B/MFN2 complex functions in BCa cells, we transfected MFN2 siRNAs in various circTAF4B context cells. The results revealed that MFN2 deficiency inhibited cell proliferation in both SW780 and J82 cells (**Figures 4A, B**) and enhanced the inhibitory effects of circTAF4B shRNA in J82 cells (**Figure 4B**). Colony formation assay manifested that MFN2 siRNAs inhibited the capability of colony formation, while circTAF4B overexpression promoted the colony formation in T24 cells (**Figure 4C**). Meanwhile, knocking down MFN2 amplified the inhibitory effects of circTAF4B shRNA on colony formation (**Figure 4D**). Flow cytometry analysis showed that MFN2 siRNAs induced G1 stage cell cycle arrest in T24 cells (**Figures 4E, F**). Knocking down circTAF4B by shRNA viral particles combined MFN2 siRNAs enhanced the cell cycle blocking in G2/M stage in J82 cells (**Figures 4G, H**). Wound healing assays demonstrated that circTAF4B promoted cell migration and that MFN2 siRNAs inhibited cell migration in T24 cells (**Figures 4I, J**) while enhancing the inhibitory effects of circTAF4B shRNA on cell migration in J82 cells (**Figures 4K, L**).

### 3.5 Anti-cancer effects of circTAF4B shRNA through MFN2-mediated AKT signaling

**Figure 5:**
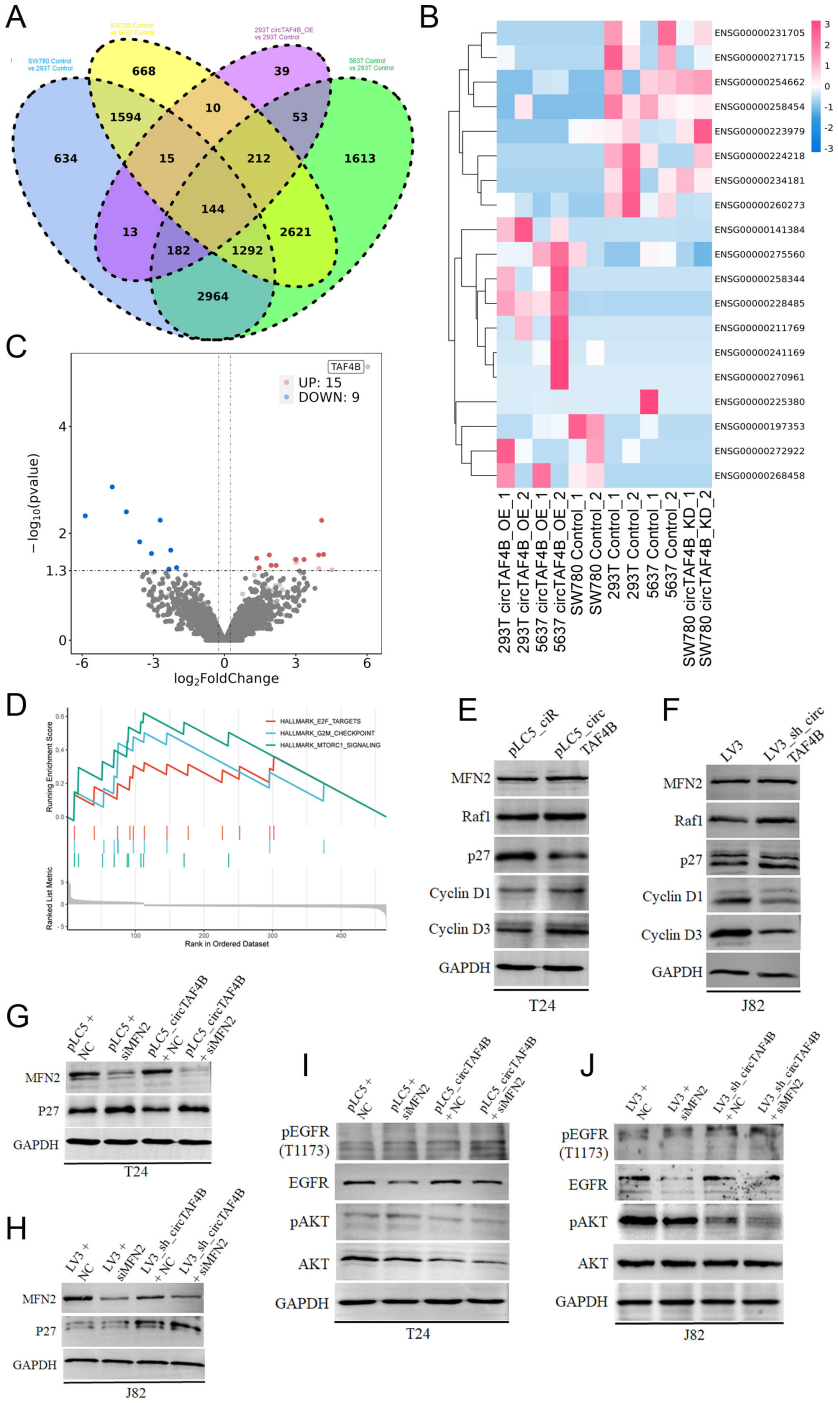
Mechanism of circTAF4B and MFN2 in BCa. **(A)** RNA-seq analysis of HEK 293T_pLC5_ciR cells and HEK 293T_pLC5_circTAF4B cells, 5637_pLC5_ciR cells and 5637_pLC5_circTAF4B cells, and SW780_LV3 cells and SW780_LV3_sh_circTAF4B cells, identifying 144 genes as overlapping sets; **(B)** Hierarchical clustering map for the top 19 genes in overlapping sets; **(C)** Volcano plot analysis comparing the circTAF4B-high group to the circTAF4B-low group; **(D)** Gene set enrichment analysis (GSEA) for the 144 overlapping genes; **(E, F)** Western blot analysis of cyclin D1, cyclin D3, p27, and MFN2 in circTAF4 overexpressed T24 cells and circTAF4 shRNA J82 cells; **(G, H)** Western blot analysis of p27 expression in circTAF4/siMFN2 and sh_circTAF4/siMFN2 contexts. **(I, J)** Western blot analysis of phosphorylation of ATK and EGFR in circTAF4/siMFN2 and sh_circTAF4/siMFN2 contexts. At least three independent western blots were conducted for **(E–J)**.

To further investigate the anti-cancer effects of circTAF4B shRNA, we conducted RNA-seq on HEK 293T cells (transfected with pLC5_ciR or pLC5_circTAF4B plasmids respectively), 5637 cells (transfected with pLC5_ciR or pLC5_circTAF4B plasmids respectively), and SW780 cells (infected with LV3 vector or LV3_sh_circTAF4B lentiviral particles respectively), and identified 144 genes as overlapping sets of the six experimental groups (**Figure 5A**). Furthermore, we analyzed the expression levels of the top 19 genes in various groups using hierarchical clustering (**Figure 5B**). Volcano plot analyses of the circTAF4B-high versus circTAF4B-low groups revealed significant upregulation of 15 genes and downregulation of 9 genes (**Figure 5C**).

GSEA for the 144 overlapping genes suggested a possible regulatory role for circTAF4B in the cell cycle and cell proliferation in BCa (**Figure 5D**). To confirm this hypothesis, we performed Western blotting on the cell cycle proteins (cyclin D1, cyclin D3, p27) and circTAF4B binding protein MFN2 under circTAF4-high/low context. The results showed that circTAF4B accelerated the cell cycle progression by enhancing cyclin D1 and D3 levels and suppressing CKI p27 levels (**Figures 5E, F**). Knockdown of circTAF4B combined with MFN2 deficiency significantly upregulated p27 expression, more effectively than circTAF4B shRNAs alone (**Figures 5G, H**).

Despite confirming the physical interaction between circTAF4B and MFN2, their cooperative function remained unclear. To address this, we analyzed downstream targets of MFN2 using Western blot analysis, revealing that the combination of circTAF4B shRNA and MFN2 siRNA distinctly suppressed phosphorylated AKT, suggesting that circTAF4B shRNA might exert anti-cancer effects through MFN2-mediated AKT signaling (**Figures 5I, J**).

## 4 Discussion

In recent years, advancements in RNA sequencing technology have led to the discovery of numerous circRNAs, which are now recognized as novel regulators in the modulation of tumorigenesis and aggressiveness of cancer cells (21, 22). Our prior research identified an intergenic circRNA, circTAF4B, upregulated in BCa. We established that circTAF4B could enhance the proliferation, migration, invasion, and EMT in BCa cells by regulating the miR-1298-5p/TGFA axis (20). In the current study, RNA pull-down assays coupled with mass spectrometry and Western blot analysis demonstrated that circTAF4B interacts with the MFN2 protein but does not affect its expression in BCa cell lines. Mechanistically, we confirmed that circTAF4B promotes cell cycle progression and AKT signaling *in vitro*.

CircRNAs represents a unique class of non-coding RNAs implicated in the development of diseases, particularly in various cancers including BCa, affecting such processess as proliferation, metabolism, metastasis, and invasion (23, 24). Functionally, most studies on cytoplasmic circRNAs have highlighted their role as “miRNA sponges” in tumors (25–27). For example, circTAF4B has been shown to sponge miR-1298-5p in our previous research (20). Increasing evidence suggests that circRNAs can also interact with proteins, thereby regulating transcription or splicing (28, 29). Notable examples include circNEIL3, which recruits the E3 ubiquitin ligase Nedd4L to degrade YBX1, inhibiting tumor metastasis (30); circCDYL2, which enhances colorectal cancer (CRC) migration by binding to Ezrin and promoting AKT phosphorylation through its upregulation (31); and circARID1A, which binds to IGF2BP3 protein, forming a ternary complex with SLC7A5 that boosts gastric cancer proliferation *via* the AKT/mTOR pathway (32). Additionally, circACTN4, potentially mediated by USF2, interacts with FUBP1 to facilitate breast cancer progression by upregulating MYC expression (33).

To elucidate the mechanisms underlying circTAF4B-mediated proliferation in BCa cells, we employed RNA pull-down assays that identified MFN2 as a circTAF4B binding protein. Known primarily for its role in mitochondrial dynamics and homeostasis, MFN2 also exhibits intriguing oncogenic or tumor-suppressing activities, depending on the cellular context. For instance, MFN2 has been shown to suppress rat HSC activation and proliferation *via* the PI3K-AKT pathway by targeting p-PDGFR-β during fibrosis progression (34). Furthermore, it has demonstrated inhibitory effects on tumor growth and metastasis in clear cell renal cell cancer through suppression of the EGFR signaling pathway (35).

The role of MFN2 in cancer appears to be context-dependent, which varies with the types of cancer and the molecular alterations within tumor. For example, suppression of MFN2 has been shown to enhance neural differentiation of embryonic stem cells by activating the AKT signaling pathway (36), while MFN2 inhibits hepatic stellate cell proliferation and attenuates liver fibrosis in rat models through the PI3K/AKT signaling pathway (34). The AKT pathway, a well-established oncogenic signaling cascade, regulates vital cellular processes such as proliferation, migration, metastasis, autophagy, and angiogenesis through its downstream effectors (37).

Previously, we identified circTAF4B, an intergenic circular RNA that exhibits upregulation in bladder cancer (BCa). In our current research, we have uncovered a potential role for circTAF4B as a critical modulator of cell cycle dynamics and proliferation in BCa. Our results demonstrate that circTAF4B promotes the advancement of the cell cycle by upregulating the expression of cyclins D1 and D3, and concurrently diminishing the levels of the cyclin-dependent kinase inhibitor p27. As TAF4B is a cell type-specific TBP-associated transcriptional factor, considering that TAF4B is the parent gene of circTAF4B, we suppose circTAF4B may downregulate the mRNA levels of p27, thus reducing p27 protein expression. This regulatory function of circTAF4B may have significant implications for the proliferative behavior of BCa cells. Our study presents compelling data demonstrating that the confluence of circTAF4B shRNA viral particles with MFN2 siRNA profoundly reduced the levels of phosphorylated AKT. Our data also demonstrated that knockdown of circTAF4B combined MFN2 siRNA raised p27 protein levels (**Figure 5H**) and weakened active AKT protein levels (**Figure 5J**). Given that circTAF4B physically bound MFN2, we speculate that circTAF4B/MFN2 complex may exerted stronger oncogenic activities than that of MFN2 alone, while circTAF4B shRNA attenuates its interaction capability leading to suppression of MFN2-mediated cell growth in BCa cells. These significant findings intimate that the therapeutic potential of circTAF4B shRNA may be realized, at least in part, through its capacity to modulate the MFN2-dependent AKT signaling pathway, thereby exerting its anti-neoplastic influence.

Our investigation has established that circTAF4B forms a physical complex with MFN2. Nonetheless, this interaction does not modulate the protein expression levels of MFN2, suggesting that the influence of circTAF4B is exerted through distinct regulatory pathways. The underlying mechanisms facilitating the collaboration between circTAF4B and MFN2 in the context of bladder cancer (BCa) are yet to be fully delineated. Consequently, our findings posit circTAF4B as a compelling target for BCa diagnosis and therapeutic intervention, potentially through its influence on MFN2 and the associated AKT signaling cascade.

## Data Availability

The original contributions presented in the study are included in the article/supplementary materials, further inquiries can be directed to the corresponding author/s.
